# Supplementary material for: Heat adaptation of phage T7 under an extended genetic code
Source: Virus Evol. 2021 Dec 1;7(2):veab100. doi: 10.1093/ve/veab100 (PMC8923235; doi:10.1093/ve/veab100)
Supplement: veab100_Supp [file veab100_supp.zip › Sequencing_Analysis.zip!/Sequencing_Analysis/A2/evidence/JC_SIDE_2_53.html]

BRESEQ :: Evidence


| New junction evidence | | | | | | | | | | | |
| --- | --- | --- | --- | --- | --- | --- | --- | --- | --- | --- | --- |
|  | | seq id | position | reads (cov) | reads (cov) | score | skew | freq | annotation | gene | product |
| \* | ? | NC\_001604.1 | = 13578 | 0 (0.000) | 7 (0.020) | 6/584 | 21.0 | 24.1% | intergenic (–/–) | *–/–* | –/– |
|  | ? | NC\_001604.1 | 14348 = | 44 (0.100) |  |  |  |  | intergenic (–/–) | *–/–* | –/– |
| Rejected: Coverage evenness skew score above cutoff. | | | | | | | | | | | |

Only 100 of 517 total aligned reads displayed.  

`CAAGACAGACTGGCTGGAAGAAACCAGAAGTTACCAAGCTGAGTCATAAGGTGTTCGATAAGGACACTATGACCCACATCAAGGCTGGTGATTGGGTTAAGGTTGACATGGGAGTTGTTGGTGGATACGGCTACGTCCGCTCAGTTAGTGGCAAATATGCACAAGTGTCATACATCACAGGTGTTACTCCACGCGGTGCAATCGTTGCCGATAAGACCAACATGATTCACACAGGTTTCTTGACAGTTGTTTCATATGAAGAGATTGTTAAGTCACGATAATCAATAGGAGAAATCAATATGATCGTTTCTGACATCGAAGCTAACGCCCTCTTAGAGAGCGTCACTAAGTTCCACTGCGGGGTTATCTACGACTACTCCACCGCTGAGTACGTAAGCTACCGTCCGAGTGACTTCGGTGCGTATCTGGATGCGCTGGAAGCCGAGGTTGCACGAGGCGGTCTTATTGTGTTCCACAACGGTCACAAGTATGACGTTCCTGCATTGACCAAACTGGCAAAGTTGCAATTGAACCGAGAGTTCCACCTTCCTCGTGAGAACTGTATTGACACCCTTGTGTTGTCACGTTTGATTC  >  NC_001604.1/14054‑14647`  

                                                                                                                                                                                                                                                                                                      `|`                                                                                                                                                                                                                                                                                                             
`accggatggcttgaaccatcaagttactcaggggaagaagagtcacactcagagtcaacagactggtccaacgacactgacttctgacaggattcttgatgactttccagacgactacgagaagtttcgctggagagtcccattctaatacgactaactaaaggagacacaccatgttcaaactgattaagaagttaggccaactgctggttcgtatgtacaacgtggaagccaagcgactgaacgatgaggctcgtaaagaggccacacagtcacgATAATCAATAGGAGAAATCAatat                                                                                                                                                                                                                                                                                                       <  1:579385‑M2/24‑1 (MQ=255)`  
`accggatggcttgaaccatcaagttactcaggggaagaagagtcacactcagagtcaacagactggtccaacgacactgacttctgacaggattcttgatgactttccagacgactacgagaagtttcgctggagagtcccattctaatacgactaactaaaggagacacaccatgttcaaactgattaagaagttaggccaactgctggttcatatgtacaacgtggaagccaagcgactgaacgatgaggctcgtaaagaggccacacagtcacgATAATCAATAGGAGAAATCAatat                                                                                                                                                                                                                                                                                                       <  1:189596‑M2/24‑1 (MQ=255)`  
   `ctacgccaactatctgatactattattgcccttgagcgtaatcagcaaggcgatatgcctaaccttgtcctcgttcgtattctcaagtgccgctttactggtgatactggtatcgatggctacatggaatacaacaaggaaaccggatggcttgaaccatcaagttactcaggggaagaagagtcacactcagagtcaacagactggtccaacgacactgacttctgacaggattcttgaCAGTTGTTTCATATGAAGAGATTGTTAAGTCACGATAATCAATAGGAGAAatcaatactgt                                                                                                                                                                                                                                                                                                    >  1:243850‑M2/241‑297 (MQ=255)`  
   `acagctggcgcactacgccaactatctgatactattattgcccttgagcgtaatcagcaaggcgatatgcctaaccttgtcctcgttcgtattctcaagtgccgctttactggtgatactggtatcgctggctacatggaatacaacaaggaaaccggatggcttgaaccatcaagttactcaggggaagaagagtcactctcagagtcaacagactggtccaacgacactgacttctgaCAGTTGTTTCATATGAAGAGATTGTTAAGTCACGATAATCAATAGGAGAAATCAATAGGAt                                                                                                                                                                                                                                                                                                    <  1:401140‑M2/61‑1 (MQ=255)`  
          `aactatctgatactattattgcccttgagcgtaatcagcaaggcgatatgcctaaccttgtcctcgttcgtattctcaagtgccgctttactggtgatacttgtatcgctggctacatggaatacaacaaggaaaccggatggcttgaaccatcaagttactcaggggaagaagagtcacactcagagtcaacagactggtctaacgacactgacttctgacaggattcttgaCAGTTGTTTCATATGAAGAGATGGTTAAGGCACGATAATCAATAGGAGAAATCAATATGATCGTTTCt                                                                                                                                                                                                                                                                                             >  1:466705‑M2/234‑301 (MQ=255)`  
             `tgggctcggagatgggtataagagacaggtcacactcatagtcaacagactggtccaacgacactgacttctgacaggattcttgatgactttccagacgactacgagaagtttcgctggagagtcccattctaatacgactcactaaaggagacacaccatgttcaaactgattaagaagttaggccaactgctggttcgtatgtacaacgtggaagccaatcgactgaacgatgaggctcgtaaagaggccacacagtcacgATAATCAATAGGAGAAATCAATATGATCATTTCTGAc                                                                                                                                                                                                                                                                                          <  1:370033‑M2/37‑1 (MQ=255)`  
              `ttctgatactattattgccattgagcgtaatcagcaaggcgatatgcctaaccttgtcctcgttcgtattctcaagtgacgctttactggtgatactggtatcgctggctacatggaatacaacaaggaaaccggatggcttgaaccatcaagttactcaggggaagaagagtcacactcagagtcaacagactggtccaacgacactgacttctgacaggtttcttgaCAGTTGTTTCATATGAAGAGATTGTTAAGTAACGATAATAAATAGGAGAAATAAATATGATCGTTTCTGACa                                                                                                                                                                                                                                                                                         >  1:297306‑M2/230‑301 (MQ=255)`  
              `atctgatactattattgcccttgagcgtaatcagcaaggcgatatgcctaaccttgtcctcgttcgtattctcaagtgccgctttactggtgatactggtatcgctggctacatggaatacaacaaggaaaccggatggcttgaaccatcaagttactcaggggaagaagagtcacactcagagtcaacagactggtccaacgacacttacttctgacaggattcttgaCAGTTGTTTCATATGAAGAGATTGTTAAGTCACGATAATCAATAGTAGAAATCAATATGATCGTTTCTGACa                                                                                                                                                                                                                                                                                         >  1:503375‑M2/230‑301 (MQ=255)`  
               `ccatcaagttactcaggggaagaagagtcacactcagagtcaacagactggtccaacgacactgacttctgacaggattcttgatgactttccagacgactacgagaagtttcgctggagagtcccattctaatacgactcactaaaggagacacaccatgttcaaactgattaagaagttaggccaactgctggttcgtatgtacaacgtggaagccaagcgactgaacgatgaggctcgtaaagaggccacacagtcacgATAATCAATAGGAGAAATCAATATGATCGTTTCTGACAt                                                                                                                                                                                                                                                                                        >  1:270575‑M2/263‑301 (MQ=255)`  
                     `aatgtgtataagagacaggagtcacactcagagtcaacagactggtccaacgacactgacttctgacaggattcttgatgactttccagacgactacgagaagtttcgctggagagtcccattctaatacgactcactaaaggagacacaccatgttcaaactgattaagaagttaggccaactgctggttcgtatgtacaacgtggaagccaagcgactgaacgatgaggctcgtaaagaggccacacagtcacgATAATCAATAGGAGAAATCAATATGATCGTTTCTGACATCGAAGc                                                                                                                                                                                                                                                                                  <  1:64966‑M2/45‑1 (MQ=255)`  
                        `attattgcccttgagcgtaatcagcaaggcgatatgcctaaccttgtcctcgttcgtattctcaagtgccgctttactggtgatactggtatcgctggctacatggaatacaacaaggaaaccggatggcttgaaccatcaagttactcagtggaagaagagtcacactcagagtcaacagactggtccaacgacactgacttcttacaggattcttgaCAGTTGTTTCATATGAAGAGATTGTTAAGTCACGATAATCAATAGGAGAAATCAATATGATCGTTTCTGACATCGAAGCTaa                                                                                                                                                                                                                                                                               >  1:372987‑M2/220‑301 (MQ=255)`  
                        `attattgcccttgagcgtaatcagcaaggcgatatgcctaaccttgtcctagttcgtattctcaagtgccgctttactggtgatactggtatcgctggatacatggaatacaacaaggaaaccggatggattgaaccatcaagttactcaggggaagaagagtcacactcagagtcaacagactggtccaacgacactgacttctgacaggattcttgaCAGTTGTTTCATATGAAGAGATTGTTAAGTCACGAtaatcaataggagaaatcaatatgctgtctcttatacacatctccga                                                                                                                                                                                                                                                                               >  1:267090‑M2/220‑278 (MQ=255)`  
                              `ggggaagacgagtcacactcagagtcaacagactggtccaacgacactgacttctgacaggattcttgatgactttccagacgactacgagaagtttcgctggagagtcccattctaatacgactcactaaaggagacacaccatgttcaaactgattaagaagttaggccaactgctggttcgtatgtacaacgtggaagtcaagcgactgaacgatgaggcccgtaaagaggccacacagtcacgATAATCAATAGGAGAAATCAATATGATCGTTTCCGACATCCAAGCtaccgactt                                                                                                                                                                                                                                                                         >  1:77936‑M2/248‑297 (MQ=255)`  
                              `ggggaagaagagtcacactcagagtcaacagactggtccaacgacactgacttctgacaggattcttgatgactttccagacgactacgagaagtttcgctggcgagtcccattctaatacgactcactaaaggagacacaccatgttcaaactgattaagaagttaggccaactgctggttcgtatgtacaacgtggaagacaagcgactgaacgatgaggctcgtaaagaggccacacagtaacgATAATCAATCGGAGAACTCAATATGATCGTTTCTGCCATCGAAGCTAACGCCct                                                                                                                                                                                                                                                                         >  1:84562‑M2/248‑301 (MQ=255)`  
                              `gcccttgagcgtaatcagcaaggcgatatgcctaaccttgtcctcgttcgtattctcaagtgccgctttactggtgatactggtatcgctggctacatggaatacaacaaggaaaccggatggcttgaaccatcaagttactcagtggaagaagagtcacactcagagtcaacagactggtccaacgacactgacttctgacaggattcttgaCAGTTGTTTCATATGAAGAGATTGTTAAGTCACGATAATCAATAGGAGAAATCAATATGATCGTTTCTGACATCGAAGCTAACGACct                                                                                                                                                                                                                                                                         >  1:816046‑M2/214‑301 (MQ=255)`  
                                  `ctattattgccctttagcgtaatcagcaaggcgatatgcctaaccttgtcctcgttcgtattctcaagtgccgctttactggtgatactggtatcgctggctacatggaatacaacaaggaaaccggatggcttgaaccatcaagttactcaggggaagaagagtcacactcagagtcaacagactggcccaacgacactgacttctgaCAGTTGTTTCATATGAAGAGATTGTTAAGTCACGATAATCAATAGGAGAAATCAATATGATCGTTTCTGACATCGAAGCTAACGCCCTCTTa                                                                                                                                                                                                                                                                     >  1:334638‑M2/210‑301 (MQ=255)`  
                                   `gctcggagatgtgtataagagacaggactggtccaacgacaatgacttctgacaggattcttgatgactttccagacgactacgagaagtttcgctggagagtcccattctaatacgactcactaaaggagacacaccatgttcaaactgattaagaagttaggccaactgctggttcgtatgtacaacgtggaagccaagcgactgaacgatgaggctcgtaaagaggccacacagtcacgATAATCAATAGGAGAAATCAATATGATCGTTTCTGACATCGAAGCTAACGCCCTCTTag                                                                                                                                                                                                                                                                    <  1:525547‑M2/59‑1 (MQ=255)`  
                                   `agaagagtcacactcagagtcaacagactggtccaacgacactgacttctgacaggattcttgatgactttccagacgactacgagaagtttcgctggagagtcccattctaatacgactcactaaaggagacacaccatgttcaaactgattaagaagttaggccaactgctggttcgtatgtacaacgtggaagccaagcgactgaacgatgaggctcgtaaagaggccacacagtcacgATAATCAATAGGAGAAATCAATATGATCGTTTCTGACATCGAAGCTAACGCCCTCTTag                                                                                                                                                                                                                                                                    <  1:432217‑M2/59‑1 (MQ=255)`  
                                     `ttattgcccttgagcgtaatcagaaaggcgatatgcctaaccttgtcctcgttcgtattctcaagtgccgctttactggtgatactggtatcgctggctacatggaatacaacaaggaaaccggatggcttgaaccatcaagttactcaggggaagaagagtcacactcagagtcaacagactggtccaacgacactgacttctgaCAGTTGTTTCATATGAAGAGATTGTTAAGTCACGATAATCAATAGGAGAAATCAATATGATCGTTTCTGACATCGAAGCTAACGCCCTCTTagag                                                                                                                                                                                                                                                                  <  1:771917‑M2/95‑1 (MQ=255)`  
                                     `tgtataagagacagggcgatatgcctaaccttgtcctagttcgtattctcaagtgccgctttactggtgatactggaatcgctggctacatggaatacaacaaggaaaccggatggcttgaaccatcaagttactcaggggaagaagagtcacactcagagtcaacagactggtcaaacgacactgacttctgacaggtttcttgaCAGTTGTTTCATATGAAGAGATTGTTAAGTCACGATAATCAATAGGAGAAATCAATATGATCGTTTCTGACATCGAAGCTAACGCACTCTTagag                                                                                                                                                                                                                                                                  <  1:319872‑M2/95‑1 (MQ=255)`  
                                     `agcgtaatcagcaaggctatatgcctaaccttgtcctcgttcgtattctcaagtgccgctttactggtgatactggtatcgctggctacatggaatacaacaaggaaaccggatggcttgaaccatcaagttactcaggggaagaagagtcacactcagagtcaacagactggtccaacgacactgacttctgacaggattcttgaCAGTTGTTTCATATGAAGAGATTGTTAAGTCACGATAATCAATAGGAGAAATCAATATGATCGTTTCTGATATCGAAGCTAACGCCCTCTTagag                                                                                                                                                                                                                                                                  <  1:228327‑M2/95‑1 (MQ=255)`  
                                     `agcgtaatcagcaaggcgatatgcctaaccttgtcctcgttcgtattctcaagtgccgatttactggtgatactggtatcgctggctacatggaatacaacaaggaaaccggatggcttgaaccatcaagttactcaggggaagaagagtcacactcagagtcaacagactggtccaacgacactgacttctgacaggattcttgaCAGTTGTTTCATATGAAGAGATTGTTAAGTCACGATAATCAATTGGAGAAATCAATATGATCGTTTCTGACATCGAAGCTAACGCACTCTTagag                                                                                                                                                                                                                                                                  <  1:122802‑M2/95‑1 (MQ=255)`  
                                     `aagagtcacactcagagtcaacagactggtccaacgacactgacttctgacaggattcttgatgactttccagacgactacgagaagtttcgctggagagtcccattctaatacgactcactaaaggagacacaccatgttcaaactgattaagaagttaggccaactgctggttcgtatgtacaacgtggaagccaagcgactgaacgatgaggctcgtaaagaggccacacagtcacgATAATCAATAGGAGAAATCAATATGATCGTTTCTGACATCGAAGCTAACGCCCTATTagag                                                                                                                                                                                                                                                                  <  1:655896‑M2/61‑1 (MQ=255)`  
                                     `aagagtcacactcagagtcaacagactggtccaacgacactgacttctgacaggattcttgatgactttccagacgactacgagaagtttcgctggagagtcccattctaatacgactcactaaaggagacacaccatgttcaaactgattaagaagttaggccaactgctggttcgtatgtacaacgtggaagcaaagcgactgaacgatgaggctcgtaaagaggccacacagtcacgATAATCAATAGGAGAAATCAATATGATCTTTTCTGACATCAAAGCTAACGCCATCTTagag                                                                                                                                                                                                                                                                  <  1:737195‑M2/61‑1 (MQ=255)`  
                                     `aagagtcacactcagagtcaacagaatggtccaacgacactgacttctgacaggattcttgatgactttccagacgactacgagaagtttcgctggagagtcccattctaatacgactcactaaaggagacacaccatgttcaaactgattaagaagttaggccaactgctggttcgtatgtacaacgtggaagccaagcgactgaacgatgaggctcgtaaagaggccacacagtcacgATAATCAATAGGAGAAATCAATATGATCGTTTCTGACATCGAAGCTAACGCCCTCTTagag                                                                                                                                                                                                                                                                  <  1:730782‑M2/61‑1 (MQ=255)`  
                                     `aaaagtcacactcagagtcaacaggctggtccaacggcactgacttctgacaggattcttgatgactatccagacgactacgagaagtttcgctggagagtcccattctaatacgactcacaaaaggggacacacaatgttcaaactgattaagaagttaggccaacttctggttcgtatgtacaacgtggaagccaagcgactgaaagatggagctcgtaaagaggccacacagtcacgATAATCAATATGAAAAATCAATATGATCGTTTCTGACATCGAAGCTAACGCCCTCTTagag                                                                                                                                                                                                                                                                  <  1:149609‑M2/61‑1 (MQ=255)`  
                                       `aggtcacactcagagtcaacagactggtccaacgacactgacttctgacaggattcttgatgactttccagacgactacgagaagtttcgctggagagtcccattctaatacgactcactaaaggagacacaccatgttcaaactgattaagaagttaggccaactgctggttcgtatgtacaacgtggaagccaagcgactgaacgatgaggctcgtaaagaggccacacagtcacgATAATCAATAGGAGAAATCAATATGATCGTTTCTGACATCGAAGCTAACGCCCTCTTagagag                                                                                                                                                                                                                                                                <  1:383424‑M2/63‑1 (MQ=255)`  
                                        `gtaatcagcaaggcgatatgcctaaccttgtcctcgttcgtattctcaagtgccgctttactggtgatactggtatcgctggctacatggaatacaacaaggaaaccggatggcttgaaccatcaagttactcaggggaagaagagtcacactcagagtcaacagactggtccaacgacactgacttctgacaggattcttgaCAGTTGTTTCATATGAAGTGATTGTTAAGTCACGATAATCAATAGTAGAAATCAATATGATCGTTTCTGACATCGAAGCTAACGCCCTCTTAGAGAGc                                                                                                                                                                                                                                                               >  1:409409‑M2/204‑301 (MQ=255)`  
                                               `ctcagagtcaacagactggtccaacgacactgacttatgacaggattcttgatgactttccagacgactacgagaagtttcgatggagagtcccattctaatacgactcactaaaggagacacaccatgttcaaactgattaagaagttaggccaactgctggttcgtatgtacaacgtggaagccaagcgactgaacgatgaggctcgtaaagaggccacacagtcacgATAATCAATAGGAGAAATCAATATGATCGTTTCTGACATCGAAGCTAACGCCCTCTTAGAGAGCGTCACTa                                                                                                                                                                                                                                                        <  1:285583‑M2/71‑1 (MQ=255)`  
                                                   `ggcgatatgcctaaccttgtcctcgttcgtattctcaagtgccgctttactggtgatactggtatcgctggctacatggaatacaacaaggaaaccggatggcttgaaccatcaagttactcaggggaagaagagtcacactcagagtcaacagactggtccaacgacactgacttctgacaggattcttgaCAGTTGTTTCATATGAAGAGATTGTTAAGTCACGATAATCAATAGGAGAAATCAATATGATCGTTTCTGACATCGAAGCTAACGCCCTCTTAGAGAGCGTCACTAAGtt                                                                                                                                                                                                                                                    >  1:451989‑M2/193‑301 (MQ=255)`  
                                                   `ggcgatatgcctaaccttgtcctcgttagtattctcaagtgccgctttactggtgatactggtatcgctggctacatggaatacaacaaggaaaccggatggcttgaaccatcaagttactcaggggaagaagagtcacactcagagtcaacagactggtccaacgacactgacttctgacaggattcttgaCAGTTGTTTCATATGAAGAGATTGTTAAGTCACGATAATCAATAGGAGAAATCAATATGATCGTTTCTGACATCGAAgctaacgccctcttagagctgtctcttatcca                                                                                                                                                                                                                                                    >  1:156932‑M2/193‑287 (MQ=255)`  
                                                     `tataagagacagccttgtcctcgttcgtattctcaagtgccgctttactggtgatactggtatcgcttgctacatggaatacaacaaggaaaccggatggcttgaaccatcaagttactcaggggaagaagagtcacactcagagtcaacagactggtccaacgacactgacttctgacaggtttcttgaCAGTTGTTTCATATGAAGAGATTGTTAAGTCACGATAATCAATAGGAGAAATCAATATGATCGTTTCTGACATCGAAGCTAACGCCCTCTTAGAGAGCGTCACTAAGTTcc                                                                                                                                                                                                                                                  <  1:535710‑M2/111‑1 (MQ=255)`  
                                                       `caacagactggtccaacgacactgacttctgacaggattcttgatgactttccagacgactacgagaagtttcgctggagagtcccattctaatacgactcactaaaggagacacaccatgttcaaactgattaagaagttaggccaactgctggttcgtatgtacaacgtggaagccaagcgactgaacgatgaggctcgtaaagaggccacacagtcacgATAATCAATAGGAGAAATCAATATGATCGTTTCTGACATCGAAGCTAACGCCCTCTTAGAGAGCGTCACTAAGTTCCAc                                                                                                                                                                                                                                                <  1:106750‑M2/79‑1 (MQ=255)`  
                                                                   `atatgcctaaccttgtcctcgttcgtattctcaagtgccgctttactggtgatactggtatcgctggctacatggaatacaacaaggaaacctgatggcttgaaccatcaagttactcaggggaagaagagtcacactcagagtcaacagactggtccaacgacactgacttctgaCAGTTGTTTCATATGAAGAGATTGTTAAGTCACGATAATCAATAGGAGAAATCAATATGATCGTTTCTGACATCGAAGCTAACGCCCTCTTAGAGAGCGTCACTAAGTTCCACTGCGGGGTTATc                                                                                                                                                                                                                                    <  1:175682‑M2/125‑1 (MQ=255)`  
                                                                       `gcctaaccttgtcctcgttcgtattctcaagtgccgctttactggtgatactggtatcgctggctacatggaatacaacaaggaaaccggatggcttgaaccatcaagttactcagggtaagaagagtcacactcagagtcaacagactggtccaacgacactgacttctgaCAGTTGTTTCATATGAAGAGATTGTTAAGTCACGATAATCAATAGGAGAAATCAATATGATCGTTTCTGACATCGAAGCTAACGCCCTCTTAGAGAgcgtcactaagttcaacctgtctcttatacaca                                                                                                                                                                                                                                >  1:253344‑M2/173‑285 (MQ=255)`  
                                                                              `ggctcggagatgtgtataagagacagtgatactggtatcgctggctacatggaatacaacaaggaaaccggatggcttgaaccatcaagttactcaggggaagaagagtcacactcagagtcaacagactggtccaacgacactgacttctgacaggattcttgaCAGTTGTTTCATATGAAGAGATTGTTAAGTCACGATAATCAATAGGAGAAATCAATATGATCGTTTCTGACATCGAAGCTAACGCCCTCTTAGAGAGCGTCACTAAGTTCCACTGCGGGGTTATCTACGACTACTc                                                                                                                                                                                                                         <  1:101125‑M2/136‑1 (MQ=255)`  
                                                                              `gacttctgacaggattcttgatgactttccagacgactacgagaagtttcgctggagagtcccattctaatacgactcactaaaggagacacaccatgttcaaactgattaagaagttaggccaactgctggttcgtatgtacaacgtggaagccaagcgactgaacgatgaggatcgtaaagaggccacacagtcacgATAATCAATAGGAGAAATCAATATGATCGTTTCTGACATCGAAGCTAACGCCCTCttagagagcgtcactaagttccacctgtctcttatacacatctccta                                                                                                                                                                                                                         >  1:214389‑M2/200‑278 (MQ=255)`  
                                                                                    `ctcaagtgccgctttactggtgatactggtatcgctggctacatggaatacaacaaggaaaccggatggcttgaaccatcaagttactcaggggaagaagagtcacactcagagtcaacagactggtccaacgacactgacttctgacaggtttcttgaCAGTTGTTTCATATGAAGAGATTGTTAAGTCACGATAATCAATAGGAGAAATCAATATGATCGTTTCTGACATCGAAGCTAACGCCCTCTTAGAGAGCGTCACTAAGTTCCACTGCGGGGTTATCTACGACTACTCCACCGc                                                                                                                                                                                                                   <  1:59532‑M2/142‑1 (MQ=255)`  
                                                                                         `gagatgtgtataagagacagctggtatcgctggctacatggaatacaacaaggaaaccggatggcttgaaccatcaagttactcaggggaagaagagtcacactcagagtcaacagactggtccaacgacactgacttctgacaggattcttgaCAGTTGTTTCATATGAAGAGATTGTTAAGTCACGATAATCAATAGGAGAAATCAATATGATCGTTTCTGACATCGAAGCTAACGCCCTCTTAGAGAGCGTCACTAAGTTCCACTGCGGGGTTATCTACGACTACTCCACCGCTGAGt                                                                                                                                                                                                              <  1:304635‑M2/147‑1 (MQ=255)`  
                                                                                            `ttcttgatgactttccagactactacgagaagtttcgctggagagtcccattctaatacgactcactaaaggagacacaccatgttcaaactgattaagaagttaggccaactgctggttcgtatgtacaacgtggaagccaagcgactgaacgatgaggctcgtaaagaggccacacagtcacgATAATCAATAGGAGAAATCAATATGATCGTTTCTGACATCGAAGCTAACGCCCTCTTAGAGAGCGTCACTAAGTTACACTGCGGGGTTATCTACGACTACTCCACCGCTGAGTACg                                                                                                                                                                                                           <  1:117137‑M2/116‑1 (MQ=255)`  
                                                                                               `ctttactggtgatactggtatcgctggctacatggaatacaacaaggaaaccggatggcttgaaccatcaagttactcaggggaagaagagtcacactcagagtcaacagactggtccaacgacactgacttctgacaggattcttgaCAGTTGTTTCATATGAAGAGATTGTTAAGTCACGATAATCAATAGGAGAAATCAATATGATCGTTTCTGACATCGAAGCTAACGCCCTCTTAGAGAGCGTCACTAAGTTCCACTGCGGGGTTATCTACGACTACTCCACCGCTGAGTACGTaa                                                                                                                                                                                                        >  1:421479‑M2/149‑301 (MQ=255)`  
                                                                                                `tgatgactttccagacgactacgagaagtttcgctggagagtcccattctaatacgactcactaaaggagacacaccatgttcaaactgattaagaagttaggccaactgctggttcgtatgtacaacgtggaagccaagcgactgaacgatgaggctcgtaaagaggccacacagtcacgATAATCAATAGGAGAAATCAATATGATCGTTTCTGACATCGAAGCTAACGCCCTCTTAGAGAGCGTCACTAAGTTCCACTGCGGGGTTATCTACGACTACTCCACCGCTGAGTACGTAAg                                                                                                                                                                                                       <  1:180797‑M2/120‑1 (MQ=255)`  
                                                                                                `tcggagatgtgtataagagaaagtactggtatcgctggctacatggaatacaacaaggaaaccggatggcttgaaccatcaagttactcaggggaagaagagtcacactcagagtcaacagactggtccaacgacactgacttctgaCAGTTGTTTCATATGAAGAGATTGTTAAGTCACGATAATCAATAGGAGAAATCAATATGATCGTTTCTGACATCGAAGCTAACGCCCTCTTAGAGAGCGTCACTAAGTTCCACTGCGGGGTTATCTACGACTACTCCACCGCTGAGTACGTAAg                                                                                                                                                                                                       <  1:677385‑M2/154‑1 (MQ=255)`  
                                                                                                  `tactggtgatactggtatcgctggctacatgtaatacaacaaggaaaccgaatggcttgaaccatcaagttactcaggggaagaagagtcacactcagagtcaacagactggtccaacgacactgacttctgacaggtttcttgaCAGTTGTTTCATATGAAGAGATTGTTAAGTCACGATAATCAATAGTAGAAATCAATATGATCGTTTCTGACATCGAAGCTAACGCCCTCTTAGAGAGCGTCACTAAGTTCCACTGCGGGGTTATCTACGACTACTCCACCGCTGAGTACGTAAGCt                                                                                                                                                                                                     <  1:797578‑M2/156‑1 (MQ=255)`  
                                                                                                   `actggtgatactggtatcgctggctacatggaatacaacaaggaaaccggatggcttgaaccatcaagttactcaggggaagaagagtcacactcagagtcaacagactggtccaacgacactgacttctgacaggattcttgaCAGTTGTTTCATATGAAGAGATTGTTAAGTCACGATAATCAATAGGAGAAATCAATATGATCGTTTCTGACATCGAAGCTAACGCCCTCTTAGAGAGCGTCACTAAGTTCCACTGCGGGGTTATCTACGACTACTCCACCGCTGAGTAAGTAAGCTa                                                                                                                                                                                                    >  1:163486‑M2/145‑301 (MQ=255)`  
                                                                                                    `tgtgtataagagacagacgagaagtttcgctggagagtcccattctaatacgactcactaaaggagacacaccatgttcaaactgattaagaagttaggccaactgatggttcgtatgtacaacgtggaagccaagcgactgaacgatgaggctcgtaaagaggccacacagtcacgATAATCAATAGGAGAAATCAATATGATCGTTTCTGACATCGAAGCTAACGCCCTCTTAGAGAGCGTCACTAAGTTCCACTGCGGGGTTATCTACGACTACTCCACCACTGAGTACGTAAGCTAc                                                                                                                                                                                                   <  1:804325‑M2/124‑1 (MQ=255)`  
                                                                                                      `agttccagatgactacgagaagtttcgctggagagtcccattctaatacgtctcactaaaggagacacaccatgttcaaactgattaagaagttaggccaactgctggttcgtatgtacaacgtggaagccaagcgactgaacgatgaggctcgtaaagaggccacacagtcacgATAATCAATAGGAGAAATCTATATGATCGTTTCTGACATCGAAGCTAACGCCCTCTTAGAGAGCGTCACTAAGTTCCACTGCGGGGTTATCTACGACTACTCCACCGCTGAGTACGTAAGCTACCg                                                                                                                                                                                                 <  1:205575‑M2/126‑1 (MQ=255)`  
                                                                                                          `atactggtatcgctggctacatggaatacaacaaggaaaccggatggcttgaaccatcaagttactcaggggaagaagagtcacactcagagtcaacagactggtccaacgacactgacttctgacagtattcttgaCAGTTGTTTCATATGAATAGATTGTTAAGTCACGATAATCAATAGGAGAAATCAATATGATCGTTTCTGACATCGAAGCTAACGCCCTCTTAGAGAGCGTCACTAAGTTCCACTGCGGGGTTATCTACGACTACTCCACCGCTGAGTACGTAAGCTACCgtctg                                                                                                                                                                                             >  1:289552‑M2/138‑299 (MQ=255)`  
                                                                                                            `gacagactacgagaagtttcgctggagagtcccattctaatacgactcactaaaggagacacacaatgttcaaactgattaagaagttaggccaactgctggttcgtatgtacaacgtggaagccaagcgactgaacgatgaggctcgtaaagaggccacacagtcacgATAATCAATAGGAGAAATCAATATGATCGTTTCTGACATCGAAGCTAACGCCCTCTTAGAGAGCGTCACTAAGTTCCACTGCGGGGTTATCTACGACTACTCCACCGATGAGTACGTAAGCTACCGTCCGAg                                                                                                                                                                                           <  1:480834‑M2/132‑1 (MQ=255)`  
                                                                                                                `gtagcgctggctacatggaatacaacaaggaaaccggagggcttgaaccatcaagttactcaggggaagaagagtcacactcagagtcaacagactggtccaacgacactgacttctgacaggattcttgaCAGTTGTTTCATATGAAGAGATTGTTAAGTCACGATAATCAATAGGAGAAATCAATATGATCGTTTCTGACATCGAAGCTAACGCCCTCTTATAGAGCGTCACTAAGTTCCACTGCGGGGTTATCTACGACTACTCCACCGCTGAGTACGTAAGCTACCGTCCGAGTGAc                                                                                                                                                                                       <  1:396011‑M2/170‑1 (MQ=255)`  
                                                                                                                   `tcgctggctacatggaatacaacaaggaaaccggatggcttgaaccatcaagttactcatgggaagaagagtcacactcagagtcaacagactggtccaacgacactgacttctgacaggattcttgaCAGTTGTTTCATATGAAGAGATTGTTAAGTCACGATAATCAATAGGAGAAATCAATATGATCGTTTCTGACATCGAAGCTAACGCCATCTTAGAGAGCGTCACTAAGTTCCACTGCGGGGTTATCTACGACTACTCCACCGCTGAGTACGTAAGCTACCGTCCGAGTGACTTc                                                                                                                                                                                    >  1:752036‑M2/129‑301 (MQ=255)`  
                                                                                                                     `gctggctacatggaatacaacaaggaaaccggatggcttgaaccatcaagttactcaggggaagaagagtcacactcagagtcaacagactggtccaacgacactgacttctgacaggattcttgaCAGTTGTTTCATATGAAGAGATTGTTAAGTCACGATAATCAATAGGAGAAATCAATATGATCGTTTCTGACATCGAAGCTAACGCCCTCTTAGAGAGCGTCACTAAGTTCCACTGCGGGGTTATCTACGACTACTCCACCGCTGAGTACGTAAGCTACCGTCAGAGTGACTTCgg                                                                                                                                                                                  <  1:111998‑M2/175‑1 (MQ=255)`  
                                                                                                                            `acatggaatacaacaaggaaaccggatggcttgaaccatcaagttactcaggggaagaaaagtcacactcagagtcaacagactggtccaacgacactgacttctgacaggattcttgaCAGTTGTTTCATATGAAGAGATTGTTAAGTCACGATAATCAATAGGAGAAATCAATATGATCGTTTCTGACATCGAAGCTAACGCCCTCTTAGAGAGCGTCACTAAGTTCCACTGCGGGGTTATCTACGACTACTCAACCGCTGAGTACGTAAGCTACCGTCCGAGTGACTTCGGTGCGTAt                                                                                                                                                                           >  1:194034‑M2/120‑301 (MQ=255)`  
                                                                                                                             `ttcgctggagagtcccattctaatacgactcactaaaggagacacaccatgttcaaactgattaagaagttaggccaactgctggttcgtatgtacaacgtggaagccaagcgactgaacgatgaggctcgtaaagaggccacacagtcacgATAATCAATAGGAGAAATCAATATGATCGTTTCTGACATCGAAGCTAACGCCATCTTAGAGAGCGTCACTACGTTCCACTGCGGGGTTATCTACGACTACTACACCGCTGAGTACGTCAGCTACCGTCCGAGTGACTTCGGTGCGTTTc                                                                                                                                                                          >  1:427262‑M2/153‑301 (MQ=255)`  
                                                                                                                                 `tcggagatgtgtataagagacaggcttgaaccatcaagttactcaggggaagaagagtcacactcagagtcaacagactggtccaacgacactgacttctgacaggattcttgaCAGTTGTTTCATATGAAGAGATTGTTAAGTCACGATAATCAATAGGAGAAATCAATATGATCGTTTCTGACATCGAAGCTAACGCCCTCTTAGAGAGCGTCACTAAGTTCCACTGCGGGGTTATCTACGACTACTCCACCGCTGAGTACGTAAGCTACCGTCCGAGTGACTTCGGTGCGTATCTGGa                                                                                                                                                                      <  1:698827‑M2/187‑1 (MQ=255)`  
                                                                                                                                 `ctggagagtcccattctaatacgactcactaaaggagacacaccatgttcaaactgattaagaagttaggccaactgctggttcgtatgtacaacgtggaagccaagctactgaacgatgaggctcgtaaagagtccacacagtcacgATAATCAATAGGAGAAATCAATATTATCGTTTCTGACATCGAAGCTAACGCCCTCTTAGAGAGCGTCACTAAGTTCCACTGCGTGGTTATCTACGACTACTCCACCGCTGAGTACGTAAGCTACCGTCCGAGTGACTTCGGTGCGTATCTGGa                                                                                                                                                                      <  1:710026‑M2/153‑1 (MQ=255)`  
                                                                                                                                     `acaacaaggaaaccggatggcttgaaccatcaagttactcaggggaagaagagtcacactcagagtcaacagactggtccaacgacactgacttctgacaggattcttgaCAGTTGTTTCATATGAAGAGATTGTTAAGTCACGATAATCAATAGGAGAAATCAATATGATCGTTTCTGACATCGAAGCTAACGCCCTCTTAGAGAGCGTCACTAAGTTCCACTGCGGGGTTATCTACGACTACTCCACCGCTGAGTACGTAAGCTACCGTCCGAGTGACTTCGGTGCGTATCTGGAtgcc                                                                                                                                                                  >  1:784717‑M2/111‑300 (MQ=255)`  
                                                                                                                                         `tcccattctaatacgactcactaaaggagacacaccatgttcaaactgattaagaagttaggccacctgctggttcgtatgtacaacgtggaagccaagcgactgaacgatgaggctagtaaagaggccacacagtcacgATAATCAATAGGAGAAATCAATATGATCGTTTCTGACATCGAAGCTAACGCCCTCTTAGAGAGCGTCACTAAGTTCCACTGCGGGGTTATCTACGACTACTCCACCGCTGAGTACGTAAGCTACCGTACGAGTGACTTCGGTGCGTATCTGGATGCgcttg                                                                                                                                                              >  1:743085‑M2/141‑299 (MQ=255)`  
                                                                                                                                             `gaaaccggatggatagaaccatcaagttactcaggggaagaatagccacactcagagtcaaaagactggtcaaacgacaataacttatgacaggatgcttgaCAGTTGTTTCATATGAAGGGATTGTTAAGTCACGAGAAGAAATAGGAGAAATAAATATGATCGTTTCTGACATCGAAGCTAACGCCCTCTTAGAGAGCGTCACTAAGTTCCACTGCGGGGTTATCTACGACTACTCCACCGCTGAGTACGTAAGCTACCGTCCGAGTGACTTCGGTGCGTATCTGGATGCGCTAGAAGc                                                                                                                                                          <  1:473790‑M2/199‑1 (MQ=255)`  
                                                                                                                                                        `gcttgaaccatcaagttactcaggggaagaagagtcacactcagagtcaacagactggtccaacgacactgacttctgacaggattcttgaCAGTTGTTTCATATGAAGAGATTGTTAAGTCACGATAATCAATAGGAGAAATCAATATGATCGTTTCTGATATCGAAGCTAACGCCCTCTTAGAGAGCGTCACTAAGTTCCACTGCGGGGTTATCTACGACTACTCCACCGCTGAGTACGTAAGCTACCGTCCGAGTGACTTCGGTGCGTATCTGGATGCGCTGGAAGCCGAGGTTGCAc                                                                                                                                               <  1:56972‑M2/210‑1 (MQ=255)`  
                                                                                                                                                           `cactaaaggagacacaccatgttcaaactgattaagaagttaggccaactgctggttcgtatgtacaacgtgtaagccaagcgactgaacgatgaggctcgtaaagaggccacacagtcccgATAATCAATAGGAGAAATCAATATGTTCGTTTCTGACATCGAAGCTAACGACCTCTTAGAGTGCGTCACTAAGTTCCACTGCGGGGTTATCTACGACTACTCCACCGCTGAGTACGTAAGCTACCGTCCGAGTGACTTCGGTCCGTATCTGGATGCGCTGGAAGCCGAGGTTGCACGAg                                                                                                                                            >  1:610839‑M2/123‑301 (MQ=255)`  
                                                                                                                                                           `cactaaaggagacacaccatgttcaaactgattaagaagttaggccaactgctggttcgtatgtacaacgtggaagccaagcgactgaacgatgaggctcgtaaagaggccacacagtcacgATAATCAATAGGAGACATCAATATGATCGTTTCTGACATCGAAGCTAACGCCCTCTTAGAGAGCTTCACTAAGTTCCACTGCGGGGTTATCTACGACTACTCCACCGCTGAGTACGTAAGCTACCGTCCGAGTgactttggtgcgtatctggatgcgcctgtctcttatacacatctcc                                                                                                                                            >  1:329705‑M2/123‑280 (MQ=255)`  
                                                                                                                                                                  `ggagacacaccatgttcaaactgattaagaagttaggccaactgctggttcgtatgtacaacgtggaagccaagcgactgaacgatgaggttcgtaaagaggccacacagtcacgCTAATCAATAGGAGAAGTCAATATGATCGTTTCTGACATCGAAGCTAACGCCATCTTAGAGAGCGTCACTAAGTTCCACTGCGGGGTTATCTACGACTACTCCACCGCTGCTTACGTAAGCTACCGTCCGAGTGACTTCGGTGCGTATCTGGATGCGCTGGAAGCCGAGGTTGCACGAGGCGGTCt                                                                                                                                     >  1:704880‑M2/116‑301 (MQ=255)`  
                                                                                                                                                                     `ggggctcggagatgtgtataagagacagcagagtcaacagactggtccaacgacactgacttctgacaggattcttgaCAGTTGTTTCATATGAAGAGATTGTTAAGTCACGATAATCAATAGGAGAAATCAATATGATCGTTTCTGACATCGAAGCTAACGCCCTCTTAGAGAGCGTAACTAAGTTCAACTGCGGGGTTATCTACGACTACTCCACCGCTGAGTACGTAAGCTACCGTCCGAGTGACTTCGGTGCGTATCTGGATGCGCTGGAAGCCGAGGTTGCACGAGGCGGTCTTAt                                                                                                                                  <  1:726032‑M2/223‑1 (MQ=255)`  
                                                                                                                                                                       `atgtgtataagagacaggattaagaagttaggccaactgctggttcgtatgtacaacgtggaagccaagcgactgaacgatgaggctcgtaaagaggccacacagtcacgATAATCAATAGGAGAAATCAATATGATCGTTTCTGACATCGAAGCTAACGCCCTCTTAGAGAGCGTCACTAAGTTCCACTGCGGGGTTATCTACGACTACTCCACCGCTGAGTACGTAAGCTACCGTCCGAGTGACCTCGGTGCGTATCTGGATGCGCTGGAAGCCGAGGTTGCACGAGGCGGTCTTATtg                                                                                                                                <  1:93601‑M2/191‑1 (MQ=255)`  
                                                                                                                                                                       `aagacagttttcaaactgattaagaagttaggccaacttctggttcgtatgtacaactttgaagccaagcgactgaacgatgaggctcgtaaagatgccacacagtcacgATAATCTATATGAGAAATCAATATGATCGTTTCTGACATCGAAGCTAACGCCCTCTTAGAGAGCGTCACTAAGTTCCACTGCGGGGTTATCTACGACTACTCCACCGCTGAGTACGTAAGCTACCGTCCGAGTGACTTCGGTGCGTATCTGGATGCGCTGGAAGCCGAGGTTGCATGAGGCGGTCTTATtg                                                                                                                                <  1:43803‑M2/191‑1 (MQ=255)`  
                                                                                                                                                                               `gggaagaagagtaacactcagagtcaacagactggtccaacgacactgacttctgacaggattcttgaCAGTTGTTTCATATGAAGAGATTGTTAAGTCACGATAATCAATAGGAGAAATCAATATGATCGTTTCTGACATCGAAGCTAACGCCCTCTTAGAGAGCGTCACTAAGTTCCACTGCGGGGTTATCTACGACTACTCCACCGCTGAGTACGTAAGCTACCGTCCGAGTGACTTCGGTGCGTATCTGGATgcgctggaagccgaggttgcacgctgtctcttatacacatctccg                                                                                                                        >  1:463266‑M2/69‑279 (MQ=255)`  
                                                                                                                                                                                  `caaactgattaagaagttaggccaactgctggttcgtatgtacaacgtggaagccaagcgactgaacgatgaggctcgtaaagaggccacacagtcacgATAATCAATAGGAGAAATCAATATGATCGTTTCTGACATCGAAGCTAACGCCCTCTTAGAGAGCGTCACTAAGTTCCACTGCGGGGTTATCTACGACCACTCCACCGCTGAGTACGTAAGATACCATCCGAGTGACTTCGGTGCGTATCTGGATGCGCTGGAAGCCGAGGTTGCACGAGGCGGTCTTATTGTGTTCCACAAc                                                                                                                     <  1:360683‑M2/202‑1 (MQ=255)`  
                                                                                                                                                                                  `aagaagagtcacactcagagtcaacagactggtccaacgacactgacttctgacaggattcttgaCAGTTGTTTCATATGAAGAGATTGTTAAGTCACGATAATCAATAGGAGAAATCAATATGATCGTTTCTGACATCGAAGCTAACGCCCTCTTAGAGAGCGTCACTAAGTTCCACTGCGGGGTTATCTACGACTACTACACCGCTGAGTACGTAAGCTACCGTCCGAGTGACTTCGGTGCGTATCTGGATGCGCTGGAAGCCGAGGTTGCACGAGGCGGTCTTATTGTGTTCCACAAc                                                                                                                     >  1:236187‑M2/66‑301 (MQ=255)`  
                                                                                                                                                                                         `attaagaagttaggccaactgctggttcgtatgtacaacgtggaagccaagcgactgaacgatgaggctcgtaaagaggccacacagtcacgATAATCAATAGGAGAAATCAATATGATCGTTTCTGACATCGAAGCTAACGCCCTCTTAGAGAGCGTCACTAAGTTCCACTGCGGGGTTATCTACGACTACTCCACTGCTGAGTACGTAAGCTACCGTCCGAGTGACTTCGGTGCGTATCTGGATGCGCTGGAAGCCGAGGTTGCACGAGGCGGTCTTATTGTGTTCCACAACGGTcaca                                                                                                              <  1:498088‑M2/209‑1 (MQ=255)`  
                                                                                                                                                                                         `attaagaagttaggccaactgctggttcgtatgtacaacgtggaagccaagcgactgaacgatgaggctcgtaaagaggccacacagtcacgATAATCAATAGGAGAAATCAATATGATCGTTTCTGACATCGAAGCTAACGCCCTCTTAGAGAGCGTCACTAAGTTCCACTGCGGGGTTATCTACGACTACTCCACCGCTGAGTACGTAAGCTACCGTACGAGTGACTTCGGTGCGTATCTGGATGCGCTGGAAGCCGAGGTTGCACGAGGCGGTCTTATTGTGTTCCACAACGGTcaca                                                                                                              >  1:541445‑M2/93‑301 (MQ=255)`  
                                                                                                                                                                                                `agttaggccaactgctggttcgtatgtacaacgtggaagccaagcgactgaacgatgaggctcgtaaagaggccacacagtcacgATAATCAATAGGAGAAATCAATATGATCGTTTCTGACATCGAAGCTAACGCCCTCTTAGAGATCGTCACTAAGTTCCACTGCGGGGTTATCTACGACTACTCCACCGCTGAGTCCGTAAGCTACCGTCCGAGTGACTTCGGTGCGTATCTGGAGGCGCTGGAAGCCGAGGTTGCACGAGGCGGTCTTATTGTGTTCCAAAACGGTCACAAGTATGa                                                                                                       >  1:169556‑M2/86‑301 (MQ=255)`  
                                                                                                                                                                                                 `cagagtcaacagactggtccaacgacactgacttctgacagtattcttgaCAGTTGTTTCATATGAAGAGATTGTTAAGTCACGATAATCAATAGGAGAAATCAATATGATCGTTTCTGACATCGAAGCTAACGCCCTCTTAGAGAGCGTCACTAAGTTCCACTGCGGGGTTATCTACGACTACTCCACCGCTGAGTACGTAAGCTACAGTCTGAGTGACTTCGGTGCGTATCTGGATGCGCTGGAAGCCGaggttgcacgaggcggtcttattgtgtctgtctcttatacacatctccga                                                                                                      >  1:762856‑M2/51‑278 (MQ=255)`  
                                                                                                                                                                                                     `gtcaacagactggtccaacgacactgacttctgacaggattcttgaCAGTTGTTTCATATGAAGAGATTGTTAAGTCACGATAATCAATAGGAGAAATCAATATGATCGTTTCTGACATCGAAGCTAACGCCCTCTTAGAGAGCGTCACTAAGTTCCACTGCGGGGTTATCTACGACTACTCCACCGCTGAGTACGTAAGCTACCGTCCGAGTGACTTCGGTGCGTATCTGGATGCGCTggaagccgaggtttcacgaggcggtcttattgctgtctcttctacacatctccgaccccacc                                                                                                  >  1:807533‑M2/47‑271 (MQ=255)`  
                                                                                                                                                                                                          `ctcagagtcaacagactggtccaacgacactGATTTC‑TGACAGTTGTTTCATATGAAGAGATTGTTAAGTCACGATAATCAATAGGAGAAATCAATATGATCGTTTCTGACATCGAAGCTAACGCCCTCTTAGAGAGCATCACTAAGTTCCACTGCGGGGTTATCTACGACTACTCCACCGCTGAGTACGTAAGCTACCGTCCGAGTGACTTCGGTGCGTATCTGGATGCGCTGGAAGCCGAGGTTGCACGAGGCGGTCTTATTGTGTTCCACAACGGTCACAAGTATGACGTTCCTGCAt                                                                                            <  1:88141/275‑1 (MQ=255)`  
                                                                                                                                                                                                           `tcgtgggctcggagatgtgtataagagacaggacttctgaCAGTTGTTTCATATGAAGAGATTGTTAAGTCACGATAATCAATAGGAGAAATCAATATGATCGTTTCTGACATCGAAGCTAACGCCCTCTTAGAGAGCGTCACTAAGTTCCACTGCGGGGTTATCTACGACTACTCCACCGCTGAGTACGTAAGCTACCGTCCGAGTGACTTCGGTGCGTATCTGGATGCGCTGGAAGCCGAGGTTGCACGAGGAGGTCTTATTGTGTTCCACAACGGTCACAAGTATGACGTTCCTGCAt                                                                                            <  1:629319‑M2/261‑1 (MQ=255)`  
                                                                                                                                                                                                           `aagagacagcgtatgtacaacgtggaagccaagcgactgaacgatgaggctcgtaaagaggccacacagtcacgATAATCAATAGTAGAAATCAATATGATCGTTTCTGACATCGAAGCTAACGCCCTCTTAGAGAGCGTCACTAAGTTCCACTGCGGGGTTATCTACGACTACTCCACCGCTGAGTACGTAAGCTACCGTCCGAGTGACTTCGGTGCGTATCTGGATGCGCTGGAAGCCGAGGTTGCACTAGGCGGTCTTATTGTGTTCCACAACGGTCACAAGTATGACGTTCCTGCAt                                                                                            <  1:511609‑M2/227‑1 (MQ=255)`  
                                                                                                                                                                                                                `ggttcgtatgtacaacgtggaagccaagcgactgaacgatgaggctcgtaaagaggccacacagtcacgATAATCAATAGGAGAAATCAATATGATCGTTTCTGACATCGAAGCTAACGCCCTCTTAGAGAGCGTCACTAAGTTCCACTGCGGGGTTATCTACGACTACTCCACCGCTGAGTACGTAAGCTACCGTCCGAGTGACTTCGGTGCGTATCTGGATGCGCTGGAAGCCGAGGTTGCACGAGGCGGTCTTATTGTGttccacaacggtcacaagtactgtctcttatacacatct                                                                                       >  1:662776‑M2/70‑282 (MQ=255)`  
                                                                                                                                                                                                                   `caacagactggtccaacgacactgacttctgaCAGTTGTTTCATATGAAGAGATTGTTAAGTCACGATAATCAATAGGAGAAATCAATATGATCGTTTCTGACATCGAAGCTAACGCCCTCTTAGAGAGCGTCACTAAGTTCCACTGCGGGGTTATCTACGACTACTCCACCGCTGAGTACGTAAGCTACCGTCCGAGTGACTTCGGTGCGTTTCTGGATGCGCTGGAAGCCGAGGTTGCACGAGGCGGTCTTATCGTGTTCCACAACGGCAACAAGTATGACGTTCCTGCATTGACCaaa                                                                                    >  1:545206‑M2/33‑301 (MQ=255)`  
                                                                                                                                                                                                                               `cgtggaagccaagcgactgaacgatgaggctcgtaaagaggccacacagtcacgATAATCAATAGGAGAAATCAATATGATCGTTTCTGACATCGAAGCTAACGCCCTCTTAGAGAGCGTCACTAAGTTCCACTGCGGGGTTATCTACGACTACTCCACCGCTGAGTACGTAAGCTACCGTCCGAGTGACTTCGGTGCGTATCTGGATGCGCTGGAAGCCGAGGTTGCACGAGGCGGTCTTATTGTGTTCCACAACGGTCACAAGTATGACGTTCCTGCATTGACCAAACTGGCAAAgttt                                                                        >  1:387148‑M2/55‑300 (MQ=255)`  
                                                                                                                                                                                                                                      `gccaagcgactgaacgatgaggctcgcaaagaggccacacagtcacgATAATCAATAGGAGAAATCAATATGATCGTTTCTGACATCGAAGCTAACGCCCTCTTAGAGAGCGTCACTAAGTTCCACTGCGGGGTTATCTACGACTACTCCACCGCTGAGTACGTAAGCTACCGTCCGAGTTACTTCGGTGCGTATCTGGATGCGCTGGAAGCCGAGGTTGCACGAGGCGGTCTTATTGTGTTCCACAACGGTCACAAGTATGACGTTCCTGCATTGACCAAACTGGCAAAGTTGCAATTGa                                                                 >  1:230587‑M2/48‑301 (MQ=255)`  
                                                                                                                                                                                                                                          `agcgactgaacgatgaggctcgtaaagaggccacacagtcaggATAATCAATAGGAGAAATCAATATGATCGTTTCTGACATCGAAGCTAACGCCCTCTTAGAGAGCGTCACTAAGTTCCACTGCGGGGTTATCTACGACTACTCCACCGCTGAGTACGTAAGCTACCGTCCGAGTGACTTCGGTGCGTATCTGGATGCGCTGGAAGCCGAGGTTGCACGAGGCGGTCTTATTGTGTTCCACAACGGTCACAAGTATGACGTTCCTGCATTGACCAAACTGGCAAAGTTGCAATTGAACCg                                                             <  1:555343‑M2/258‑1 (MQ=255)`  
                                                                                                                                                                                                                                                `tgaCAGTTGTTTCATATGAAGAGATTGTTAAGTCACGATAATCAATAGGAGAAATCAATATGATCGTTTCTGACATCGAAGCTAACGCCCTCTTAGAGAGCGTCACTAAGTTCCACTGCGGGGTTATCTACGACTACTCCACCGCTGAGTACGTAAGCTACCGTCCGAGTGACTTCGGTGCGTATCTGGATGCGCTGGAAGCCGAGGTTGCACGAGGCGGTCTTATTGTGTTCAACAACGGTCACAAGTATGACGTTCCTGCATTGACTAAACTGGCAAAGTTGCAATTGAACCGAGAGtt                                                       >  1:519005‑M2/4‑301 (MQ=255)`  
                                                                                                                                                                                                                                                      `gtgggccggagatgtgtataagagacagacgATAATCAATAGGAGAAATCAATATGATCGTTTCTGACATCGAAGCTAACGCCCTCTTAGAGAGCGTCACTAAGTTCCACTGCGGGGTTATCTACGACTACTCCACCGCTGAGTACGTAAGCTACCGTCCGAGTGACTTCGGTGCGTATCTGGATGCGCTGGAAGCCGAGGTTGCACGAGGCGGTCTTATTGTGTTCCACAACGGTCACAAGTATGACGTTCCTGCATTGACCAAACTGGCAAAGTTGCAATTGAACCGAGAGTTCCACCt                                                 <  1:493737‑M2/270‑1 (MQ=255)`  
                                                                                                                                                                                                                                                            `cATATGAAGAGATTGTTAAGTCACGATATTCAATAGGAGAAATCAATATGATCGTTTCTGACATCGAAGCTAACGCCCTCTTAGAGAGCGTCACTAAGTTCCACTGCGTGGTTATCTACGACTACTCCACCGCTGAGTACGTAAGCTACCGTCCGAGTGACTTCGGTGCGTATCTGGATGCGCTGGAAGCCTAGGTTGCACGAGGCGGTCTTATTGTGTTCCACAACGGTCACAAGTATGACGTTCCTGCATTGACCAAACTGGCAAAGCTgtctcttatacacatctccgagcccacgag                                           >  1:417743/1‑272 (MQ=255)`  
                                                                                                                                                                                                                                                                 `aaggaggcaaaaaaGTAAAGATAAACAATAGGAGAAATCAATATGATCGTTTCTGACATCGAAGCTAACGCAATAATAGAGAGCGTCACTAAGTTCGACTGCGGGGTTATCTACGACGACTCCACCGCAGAGTACGTAAGCTACCGTCCGAGTGAATTAGGTGCGTAAATGGATGCGCTGGAAGAGGAGGTTGCACGAGGCGGTCTTATAGTGTTACACAACGGTCACAAGTATGACGTTCCTGCATTGACCAAACTGGCAAAGTTGCAATTGAACCGAGAGTTCCACCTTCCTCGTgaga                                      <  1:605913/289‑1 (MQ=255)`  
                                                                                                                                                                                                                                                                   `agaggccacacagtcacgATAATCAATAGGAGAAATCAATATGATCGTTTCTGACATCGAAGCTAACGCCCTCTTAGAGAGCGTCACTAAGTTCCACTGCGGGGTTATCTACGACTACTCCACCGCTGAGTACGTAAGCTACCGTCCGAGTGACTTCGGTGCGTATCTGGATGCGCTGGAAGCCGAGGTTGCACGAGGCGGTCTTATTGTGTTCCACAACGGTCACAAGTATGACGTTCCTGCATTGACCAAACTGGCAAAGTTGCAATTGAACCGAGAGTTCCACCTTCCTCGTGAGAAc                                    <  1:263021‑M2/283‑1 (MQ=255)`  
                                                                                                                                                                                                                                                                    `gagcTTGTTAAGTCACGATAATCAATAGGAGAAATCTATATGATCGTTTCTGACATCGAAGCTAACGCCCTCTTAGAGAGCGTCACTAAGTTCCACTGCGGGGTTATCTACGACTACTCCACCGCTGAGTACGTAAGCTACCGTCCGAGTGACTTCGGTGCGTATCTGGATGCGCTGGAAGCCGAGGTTGCACGAGGCGGTCTTATTGTGTTACACAACGGTCACAAGTATGACGTTCCTGCATTGACCAAACTGGCAAAGTTGCAATTGAACCGAGAGTTCCACCTTCCTCGTGAGAACt                                   >  1:256094/1‑301 (MQ=255)`  
                                                                                                                                                                                                                                                                      `ggccacacagtcacgATAATCAATAGGAGAAATCAATATGATCGTTTCTGACATCGAAGCTAACGCCCTCTTAGAGAGCGTCACTAAGTTCCACTGTGGGGTTATCTACGACTACTCCACCGCTGAGTACGTAAGCTACCGTCCGAGTGACTTCGGTGCGTATCTGGATGCGCTGGAAGCCGAGGTTGCACGAGGCGGTCTTATTGTGTTCCACAACGGTCACAAGTATGACGTTCCTGCATTGACCAAACTGGCAAAGTTGCAATTGAACCGAGAGTTCCACCTtcctcgtgactgtccc                                 >  1:747704‑M2/16‑294 (MQ=255)`  
                                                                                                                                                                                                                                                                      `ggccacacagtcacgATAATCAATAGGAGAAATCAATATGATCGTTTCTGACATCGAAGCTAACGCCCTCTTAGAGAGCGTCACTAAGTTCCACTGCGGGGTTATCTACGACTACTCCACCGCTGAGTACGTAAGCTACCGTCCGAGTGACTTCGGTGCGTATCTGGATGCGCTGGAAGCCGAGGTTGCACGAGGCGGTCTTATTGTGTTCCACAACGGTCACAAGTATGACGTTCCTGCATTGACCAAACTGGCAAAGTTGCAATTGAACCGAGAGTTCCACCTTCCTCGTGAGAACTGt                                 >  1:348852‑M2/16‑301 (MQ=255)`  
                                                                                                                                                                                                                                                                        `ttGTTAAGTCACGATAATCAATAGGAGAAATCAATATGATCGTTTCTGACATCGAAGCTAACGCCCTCTTAGAGAGCGTCACTAAGTTCCACTGCGGGGTTATCTACGACTACTCCACCGATGAGTACGTAAGCTACCGTCCGAGTGACTTCGGTGCGTATCTGGATGCGCTGGAAGCCGAGGTTGCACGAGGCGGTCTTATTGTGTTCCACAACGGTCACAAGTATGACGTTCCTGCATTGACCAAACTGGCAAAGTTGCAATTGAACCGAGAGTTCCACCTTCCTCGTGAGAACTGTAt                               <  1:755798/301‑1 (MQ=255)`  
                                                                                                                                                                                                                                                                             `aaGTCACGATAATCAATAGGAGAAATCAATATGATCGTTTCTGACATCGAAGCTAACGCCCTCTTAGAGAGCGTCACTAAGTTCCACTGCGGGGTTATCTACGACTACTCCACCGCTGAGTACGTAAGCTACCGTCCGAGTGACTTCGGTGCGTATCTGGATGCGCTGGAAGCCGAGGTTGCACGAGGCGGTCTTATTGTGTTCCACAACGGTCACAAGTATGACGTTCCTGCATTGACCAAACTGGAAAAGTTGCAATTGAACCGAGAGTTCCACCTTCATCGTGAGAACTGTATTGaca                          <  1:126979/301‑1 (MQ=255)`  
                                                                                                                                                                                                                                                                                   `cgATAATCAATAGGATAAATCAATATGATCGTTTCTGACATCGAAGCTAACGCCCTCTTAGAGAGCGTCACTAAGTTCCACTGCGGGGTTATCTACGACTACTCCACCGCTGAGTACGTAAGCTACCGTCCGAGTGACTTCGGTGCGTATCTGGATGCGCTGGAAGCCGAGGTTGCACGAGGCGGTCTTATTGTGTTCCACAACGGTCACAAGTATGACGTTCCTGCATTGACCAAACTGGCAAAGTTGCAATTTAACCGAGAGTTCCACCTTCCTCGTGAGAACTGTATTGACACCCTtg                    <  1:581354‑M2/299‑1 (MQ=255)`  
                                                                                                                                                                                                                                                                                        `aTCAATAGGAGAAATCAATATGATCGTTTCTGACATCGAAGCTAACGCCCTCTTAGAGAGCGTCACTAAGTTCCACTGCGGGGTTATCTACGACTACTCCACCGCTGAGTACGTAAGCTACCGTCCGAGTGACTTCGGTGCGTATCTGGATGCGCTGGAAGCCGAGGTTGCACGAGGCGGTCTTATTGTGTTCCACAACGGTCACAAGTATGACGTTCCTGCATTGACCAAACTGGCAAAGTTGCAATTGAACCGAGAGTTCCACCTTCCTCGTTAGAACTGTATTGACACCCTTGtgttg               >  1:587121/1‑301 (MQ=255)`  
                                                                                                                                                                                                                                                                                          `cAATAGTAGAAATCAATATGATCGTTTCTGACATCGAAGCTAACGCCCTCTTAGAGAGCGTCACTAAGTTCCACTGCGGGGTTATCTACGACTACTCCACCGCTGAGTACGTAAGCTACCGTCCGAGTGACTTCGGTGCGTATCTGGATGCGCTGGAAGCCGAGGTTGCACGAGGCGGACTTATTGTGTTCCACAACGGTCACAAGTATGACGTTCCTGCATTGACCAAACTGGCAAAGTTGCAATTGAACCGAGAGTTCCACCTTCCTCGTGAGAACTGTATTGACACCCTTGTGTTGTc             >  1:353015/1‑301 (MQ=255)`  
                                                                                                                                                                                                                                                                                          `cAATAGGAGAAATCAATATGATCGTTTCTGACATCGAAGCTAACGCCCTCTTAGAGAGCGTCACTAAGTTCCACTGTGGGGTTATCTACGACTACTCCACCGCTGAGTACGTAAGCTACCGTCCGAGTGACTTCGGTGCGTATCTGGATGCGCTGGAAGCCGAGGTTGCACGAGGCGGTCTTATTGTGTTCCACAACGGTCACAAGTATGACGTTCCTGCATTGACCAAACTGGCAAAGTTGCAATTGAACCGAGAGTTCCACCTTCCTCGTGCGAACTGTATTGACACCCTTGtgttggc             >  1:448673/1‑299 (MQ=255)`  
                                                                                                                                                                                                                                                                                          `aaaTAGGAGAAATCAATATGATCGTTTCTGACATCGAAGCTAACGCCCTCTTAGAGAGCGTCACTAAGTTCCACTGCGGGGTTATCTACGACTACTCCACCGCTGAGTACGTAAGCTACCGTCCGAGTGACTTCGGTGCGTATCTGGATGCGCTGGAAGCCGAGGTTGCACGAGGCGGTCTTATTGTGTTCCACAACGGTCACAAGTATGACGTTCCTGCATTGACCAAACTGGCAAAGTTGCAATTGAACCGAGAGTTCCACCTTCCTCGTGAGAACTGTATTGACACCCTTGTGTTGTc             <  1:139863/300‑1 (MQ=255)`  
                                                                                                                                                                                                                                                                                           `aaTAGGAGAAATCAATAGGATCGTTTCTGACATCGAAGCTAACGCCCTCTTAGAGAGCGTCACTAAGTTCCACTGCGGGGTTATCTACGACTACTCCACCGCTGAGTACGTAAGCTACCGTCCGAGTGACTTCGGTGCGTATCTGGATGCGCTGGAAGCCGAGGTTGCACGAGGCGGTCTTATTGTGTTCCACAACGGTCACAAGTATGACGTTCCTGCATTGACCAAACTGGCAAAGTTGCAATTGAACCGAGAGTTCCACCTTCCTCGTGAGAACTGTATTGACACCCTTGTGTTGTCa            <  1:822486/301‑1 (MQ=255)`  
                                                                                                                                                                                                                                                                                            `atatGAGACATCAATATGATCGTTTCTGACATCGAAGCTAACGCCCTCTTAGAGAGCGTAACTAAGTTCCACTGCGGGGTTATCTACGACTACTCCACCGCTGAGTACGTAAGCTACCGTCCGAGTGACTTCGGTGCGTATCTGGATGCGCTGGAAGCCGAGGTTGCACGAGGCGGTCTTATTGTGTTCCACAACGGTCACAAGTATGACGTTCCTGCATTGACCAAACTGGCAAAGTTGCAATTGAACCGAGAGTTCCACCTTCCCCGTGAGAACTGTATTGACAcctgtctcttataca           >  1:637937/1‑288 (MQ=255)`  
                                                                                                                                                                                                                                                                                                    `aaTCAATATGATCGTTTCTGACATCGAAGCTAACGCCCTCTTAGAGAGCGTCACTAAGTTCCACTTCTGGGTTATCTACGACTACTCCACCGCTGAGTACGTAAGCTACCGTCCGAGTGACTTCGGTGCGTATCTGGATGCGCTGGAAGCCGAGGTTGCACGAGGCGGTCTTATTGTGTTCCACAACGGTCACAAGTATGACGTTCCTGCATTGACCAAACTGGCAAAGTTGCAATTGAACCGAGAGTTCCACCTTCCTCGTGAGAACTGTATTGacactgtctcttatacacatctccga   >  1:691598/1‑279 (MQ=255)`  

                                                                                                                                                                                                                                                                                                      `|`                                                                                                                                                                                                                                                                                                             
`CAAGACAGACTGGCTGGAAGAAACCAGAAGTTACCAAGCTGAGTCATAAGGTGTTCGATAAGGACACTATGACCCACATCAAGGCTGGTGATTGGGTTAAGGTTGACATGGGAGTTGTTGGTGGATACGGCTACGTCCGCTCAGTTAGTGGCAAATATGCACAAGTGTCATACATCACAGGTGTTACTCCACGCGGTGCAATCGTTGCCGATAAGACCAACATGATTCACACAGGTTTCTTGACAGTTGTTTCATATGAAGAGATTGTTAAGTCACGATAATCAATAGGAGAAATCAATATGATCGTTTCTGACATCGAAGCTAACGCCCTCTTAGAGAGCGTCACTAAGTTCCACTGCGGGGTTATCTACGACTACTCCACCGCTGAGTACGTAAGCTACCGTCCGAGTGACTTCGGTGCGTATCTGGATGCGCTGGAAGCCGAGGTTGCACGAGGCGGTCTTATTGTGTTCCACAACGGTCACAAGTATGACGTTCCTGCATTGACCAAACTGGCAAAGTTGCAATTGAACCGAGAGTTCCACCTTCCTCGTGAGAACTGTATTGACACCCTTGTGTTGTCACGTTTGATTC  >  NC_001604.1/14054‑14647`

  

Alignment Legend

Aligned base mismatch/match (shaded by quality score): `ATCG/ATCG` `< 3 ≤` `ATCG/ATCG` `< 15 ≤` `ATCG/ATCG` `< 27 ≤` `ATCG/ATCG` `< 37 ≤` `ATCG/ATCG` `< 38 ≤` `ATCG/ATCG`

Unaligned base: `atcg`    Masked matching base: `atcg`    Alignment gap: `‑`    Deleted base: `‑`

Reads not counted as support for junction

read\_name Not counted due to insufficient overlap past the breakpoint.

read\_name Not counted due to not crossing MOB target site duplication.
